# Significant reductions of host abundance weakly impact infection intensity of *Batrachochytrium dendrobatidis*

**DOI:** 10.1371/journal.pone.0242913

**Published:** 2020-11-30

**Authors:** Jaime Bosch, Luis M. Carrascal, Andrea Manica, Trenton W. J. Garner

**Affiliations:** 1 Research Unit of Biodiversity (CSIC, UO, PA), Gonzalo Gutiérrez Quirós s/n, Oviedo University - Campus Mieres, Edificio de Investigación, Mieres, Spain; 2 Centro de Investigación, Seguimiento y Evaluación, Rascafría, Spain; 3 Museo Nacional de Ciencias Naturales-CSIC, Madrid, Spain; 4 Department of Zoology, University of Cambridge, Cambridge, United Kingdom; 5 Institute of Zoology, Zoological Society of London, London, United Kingdom; Universitat Autonoma de Barcelona, SPAIN

## Abstract

Infectious diseases are considered major threats to biodiversity, however strategies to mitigate their impacts in the natural world are scarce and largely unsuccessful. Chytridiomycosis is responsible for the decline of hundreds of amphibian species worldwide, but an effective disease management strategy that could be applied across natural habitats is still lacking. In general amphibian larvae can be easily captured, offering opportunities to ascertain the impact of altering the abundance of hosts, considered to be a key parameter affecting the severity of the disease. Here, we report the results of two experiments to investigate how altering host abundance affects infection intensity in amphibian populations of a montane area of Central Spain suffering from lethal amphibian chytridiomycosis. Our laboratory-based experiment supported the conclusion that varying density had a significant effect on infection intensity when salamander larvae were housed at low densities. Our field experiment showed that reducing the abundance of salamander larvae in the field also had a significant, but weak, impact on infection the following year, but only when removals were extreme. While this suggests adjusting host abundance as a mitigation strategy to reduce infection intensity could be useful, our evidence suggests only heavy culling efforts will succeed, which may run contrary to objectives for conservation.

## Introduction

Managing infectious diseases is one of the great challenges of the 21^st^ Century. A growing array of pathogens impair human health, threaten food security and pose a risk to biodiversity conservation, however our efforts to control infections circulating in wildlife populations are largely unsuccessful (but see [1–5]). Developing and testing strategies for controlling infectious agents should therefore be a research priority, yet surprisingly few real-world trials of methods for controlling infectious disease affecting wildlife have been undertaken and subsequently published [1–4,6–9]. Among others, culling of hosts from a susceptible population is a fundamental approach in density-dependent disease mitigation based on the theory of disease ecology and epidemiology [10–15]. Note, however, that while the term culling often denotes euthanizing, it can simply be the removal of animals from the system as in this study (see below).

Most studies for controlling infectious agents are performed in humans, livestock or companion animals, whereas infections strictly affecting wildlife are largely overlooked, despite overwhelming evidence that wildlife diseases are behind a significant proportion of recent and ongoing biodiversity loss [16]. One exception is the case of the chytridiomycosis, a disease responsible for the decline of hundreds of amphibian species, with new reports accruing [17]. An enormous body of literature bears witness to the research that has revealed the ecological and evolutionary drivers of disease [18,19], but only a relatively sparse publication list provides recommendations on how to respond to the threat chytrid fungi pose to amphibian biodiversity [20–28]. Even more sparse is the collection of published studies reporting the outcomes of attempts at mitigating infections and disease in nature, none of which presents a scalable and transferable solution [5,29–31]. This is unfortunate: although a small proportion of species affected by chytridiomycosis appear to be recovering without intervention, most of them continue to decline [17]. Without methods to combat the disease *in situ*, it seems clear that the ‘chytrid crisis’ will continue to impair global amphibian biodiversity.

We have argued elsewhere that research should focus on approaches that are transferrable across host species and communities, easily implemented by nonspecialists and amenable to combination with other techniques [22]. The list of possible actions is short, at least in part due to the fact that techniques commonly used for controlling disease in mammals and birds, like vaccines, simply cannot be applied to most amphibian communities due to their typically large population sizes. Many species of amphibians can be easily captured or trapped, though, even when local abundance may be in the tens of thousands, which offers opportunities to ascertain the impact of altering the abundance of hosts. Several studies have argued for a potential, but as yet uncertain, role of host abundance on the prevalence and amplification of infection loads [32–34]. The latter is a key parameter affecting the severity of chytridiomycosis, as there is a strong, positive relationship between the infection intensity of *Batrachochytrium dendrobatidis* (*Bd*), one of the two pathogens causing chytridiomycosis, and the likelihood of dying from the disease [35,36].

Chytridiomycosis was first described in Europe nearly 20 years ago in the Sierra de Guadarrama mountains of Spain [37] where this study took place. At this location, *Alytes obstetricans* (midwife toads) were extirpated, and two other amphibian species were reported dying from chytridiomycosis and potentially declining as a result: *Salamandra salamandra* (fire salamanders) and *Bufo spinosus* (spiny common toads [38–40]). *Alytes obstetricans* is highly susceptible to *Bd* infection and was historically the most abundant species and primary source of chytridiomycosis [37,38]. *Salamandra salamandra* has also historically experienced lethal chytridiomycosis and sharp declines when *A*. *obstetricans* was abundant in the area, even though it is not considered to be as highly susceptible to infection and disease [39]. Larvae of both species can overwinter and are thought to act as reservoirs transmitting infections to incoming young of the year larvae [41]. The extirpation of *A*. *obstetricans* from the area means that overwintering *S*. *salamandra* larvae are considered the primary source of infection in the aquatic environment [39,42].

Because of their purported role in maintaining infections, we chose to investigate how altering host abundance of *S*. *salamandra* larvae in the most important amphibian breeding ponds in the Sierra de Guadarrama mountains affects infection intensity the following breeding season, under the assumption that disease risk is reduced at lower infection loads. Pilot experiments to establish drug concentrations to clear *Bd* infections of *S*. *salamandra* larvae indicated that most individuals lost infection when housed individually. Thus, our hypothesis was that, at least for fire salamander larvae that play a key role in intraspecific transmission but are weakly infected, significant alterations in host density could reduce strength of infections. We tested this through a large-scale, 2-year larval removal field trial in the permanent ponds where overwintering occurs. Additionally, we used an *ex situ* approach to examine how differing densities of larvae affected the intensity of infection in groups of *S*. *salamandra* and *A*. *obstetricans* larvae over time scales similar to those where infection dynamics change in the wild [41].

## Materials and methods

### Study area

**Fig 1:**
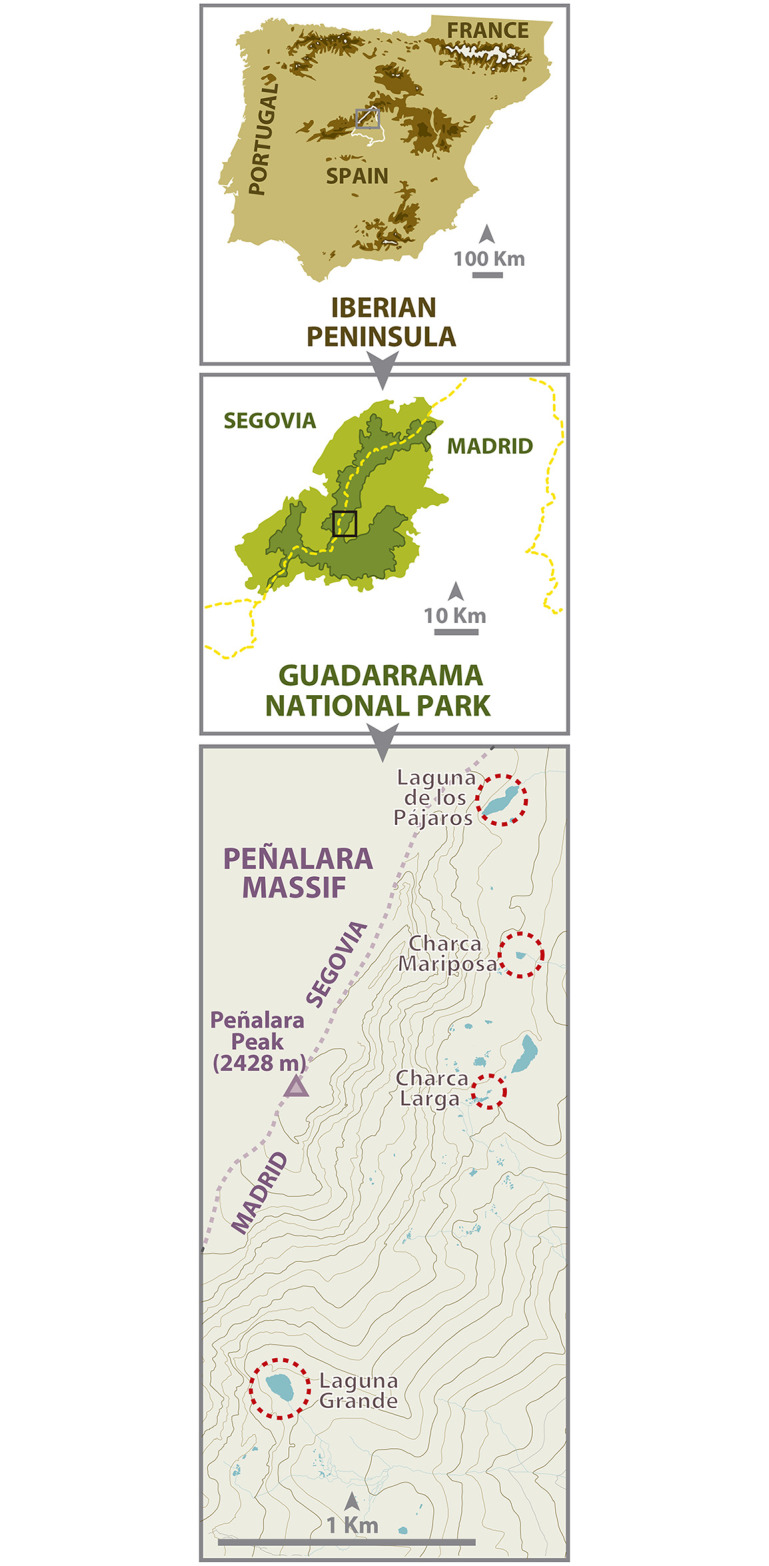
Location of the 4 study ponds for fire salamander larvae located in the Peñalara Massif within the Guadarrama National Park, Central Spain, summer 2015-spring 2018.

The field experiment and the collection of salamander larvae for the lab experiment took place in the protected area of Guadarrama Mountains National Park, central Spain (408500 N, 38570 W; Fig 1). The core of the park, the Peñalara Massif, is a wetland system of about 800 ha composed of several small glacial valleys located 1800–2430 m a.s.l. The area holds around 250 fishless ponds, mostly temporary, and supports nine species of amphibian. The landscape of this alpine area consists mainly of granitic outcrops, alpine meadows, heathlands dominated by *Cytissus oromediterraneus* and *Juniperus communis nana*, and forests of *Pinus sylvestris* below the timber line. Local climate is characterized by very cold winters, mild summers and abundant precipitation, with snow falls usually from the end of November to the middle of May. *Batrachochytrium dendrobatidis* has been present in the area for at least 20 years [37], whereas neither *B*. *salamandrivorans* nor *Ranavirus* have not been found (Bosch, unpublished results).

The field study took place from Summer 2015 to Spring 2018 in the four permanent ponds that host the largest *S*. *salamandra* populations: Laguna Grande (elevation 2018 m, surface 6766 m^2^, maximum depth 2.7 m), Charca Larga (elevation 2111 m, surface 89 m^2^, maximum depth 0.6 m), Charca Mariposa (elevation 2136 m, surface 660 m^2^, maximum depth 0.7 m) and Laguna de los Pájaros (elevation 2170 m, surface 5175 m^2^, maximum depth 1.3 m). These ponds freeze in December and thaw in March, and they are separated by at least 0.5 km. No overwintering larvae of other amphibian species are present and only *B*. *spinosus* is relatively abundant in those ponds.

### *Ex situ* assessment of the impact of host density on infection intensity

Naturally *Bd*-infected overwintering larvae of *Alytes obstetricans* and *S*. *salamandra* at the same stage of development were collected at the end of the winter, respectively, in the Teruel province (Aragón, Northeastern Spain) and in Laguna Grande (Guadarrama Mountains National Park). For both species, prevalence of *Bd* infection in larval stages is known to approach 100% during colder months at both localities [41,43]. The experiment took place in bio-secure conditions at the laboratories of the Research and Management Center ‘Puente del Perdón’, a facility of the Guadarrama Mountains National Park located a few km away of the Peñalara Massif, and where head-starting programs for *A*. *obstetricans* and *Rana iberica* are hosted. The larvae we used were returned to their point of capture at the end of the *ex situ* experiment. Initial *Bd* infections were confirmed following the qPCR protocol of Boyle et al. [44]. We housed indoors groups of larvae of each species in separate 5 L plastic tanks at three different densities (one, five and 15 individual each) on a 12:12-h light schedule. The densities ranged from the absolute minimum (one) up to the maximum amount of salamander larvae we could house in a single container without having them injuring each other, and while keeping densities consistent across species. Density treatments were replicated respectively seven, six and four times for *S*. *salamandra* and eight, six and four times for *A*. *obstetricans* (for densities of one, five and 15 larvae, respectively). We fed animals *ad libitum* with tubifex worms (salamanders) or commercial tadpole food (midwife toads), and renewed half of the water in each tub weekly. Water temperature was recorded throughout every half hour with a HOBO Water Temperature Pro v2 datalogger, and varied from 0.8 to 9.8°C. We used average water temperature during the three days prior to *Bd* sampling for analysis, following Fernández-Beaskoetxea et al. [43], who described short-term impacts of water-temperature on *Bd* load. We tracked changes in infections in one animal per tank, repeatedly sampling the same animal once at the start of the experiment and every month afterwards, for a total of five samples per animal and tank. Focal *A*. *obstetricans* tadpoles were identified using visual implant elastomers and swabbed on their keratinized mouthparts, while focal *S*. *salamandra* were identified by unique dorsal spot patterns and swabbed across the entire body surface. We quantified load of *Bd* infections (referred to as load hereafter) following Boyle et al. [44] by using cotton swabs (MW100 rayon tipped dry swabs from MWE Medical Wire). Swabs samples were kept at 4°C until analyses and the DNA was extracted with Prep-Man Ultra and amplified using a BIO-RAD CFX96 Real-Time PCR Detection System. Each 96-well assay plate included a negative control and four different standards containing DNA from 100, 10, 1 and 0.1 *Bd* zoospore equivalents (ZE). We tested all the samples (diluted 1/10), as well as the negative control and the standards, in duplicate. We considered samples with greater than 0.1 ZE in both replicates, and the expected sigmoidal shaped amplification curve, positive for *Bd*.

We used a general linear mixed models to analyze the within-subjects variation in *Bd* load. Species was treated as a categorical fixed factor, the “sampling events” (date of sampling) and the density treatment as ordered factors, and water temperature during the three days prior to sampling as a covariate in the mixed models, with tadpole identity as a random effect. Three different mixed random intercept models were built: (a) null model without any fixed effect, (b) main fixed effects model, and (c) full model containing the main effects and their interactions. Second-order Akaike’s AICc for finite sample sizes were used to compare these three models, using maximum likelihood (ML) estimation; the model with the lowest AICc figure was finally obtained by means of restricted maximum likelihood (REML) estimation, because it renders unbiased variance covariance parameters. *Bd* load (including zero values) was log10 transformed (y’ = log10[y+1]) prior to data analyses. The mean square (MS) and degrees of freedom (df) of the error terms were estimated following the Kenward-Roger method [45]. Homoscedasticity and normality of residuals of the final general linear mixed model were visually checked and did not show considerable deviations from the model assumptions. All analyses were carried out using R version 3.1.2 (R Core Team 2014) and the packages lme4 [46], lmerTest [47], lmtest [48], pbkrtest [49], car [50], MuMIn [51] and phia [52]. The proportion of variance explained by the model was obtained using the r.squaredGLMM function of the MuMIn package [53], while the estimations for different predictors were obtained using the SS (sums of squares) provided by the function anova.lmer of the lmerTest package (Partial-eta^2^ according to the ratio SS_effect_/[SS_effect_+SS_error_]).

### Field trial experiment

Four permanent ponds within the Peñalara Massif support significant larval fire salamander populations. The combination of lithology, scarcity of aquatic vegetation, low depth and crystal-clear waters is extremely favorable for larval counts and captures [38]. We removed salamander larvae from three ponds (Laguna de Pájaros, Charca Mariposa and Charca Larga) at the end of the summers of 2015 and 2016, and early spring of 2017, when no larvae of other species were present. We removed 1149 larvae from Laguna de Pájaros, 214 from Charca Mariposa and 32 from Charca Larga in 2015, 2057 larvae from Laguna de Pájaros in 2016 and 1701 larvae across all three sites in 2017. We did not remove larvae from Laguna Grande, which served as our control pond. The larvae we removed were reared in captivity in the facilities of the National Park referred to above and returned as metamorphosed juveniles to the surroundings of their pond of capture in early spring (2015 and 2016 collections) or late summer (2017 collection). Ninety-five casualties occurred as the result of removal actions, while approximately 5100 juveniles were released.

To estimate load of infection, we sampled a maximum of 20 larvae at each pond in early spring and late summer (from summer 2015 to spring 2018). We selected a sample size of 20 because our previous work in the study area and on this species showed no significant differences of load of infection among ponds within a year [41]. By definition spring samples were entirely composed of overwintering larvae, while summer samples were a mix of young of the year and overwintered animals. Again, load was quantified using qPCR as described above [44].

We estimated the removal effort (percent removed) for each removal event in each pond by counting the number of larvae before starting the removal activity and the night after. However, for statistical analyses binary values for removal activity (0: no removals, 1: removals) preceding the breeding season were assigned to each pond for each year (e.g., removal activity at the end of the summer of 2016 and at the early spring of 2017 were assigned to 2017 for analyses). *Bd* load (including zero values) was log-transformed to achieve normality and homoscedasticity of residuals. We fitted a general linear mixed model using the same procedures described above to detect differences in infection intensity between removal activity and among ponds, years and seasons. ‘Pond’ was considered a random effect, whereas ‘year’, ‘season’ and ‘removal activity’ were considered as fixed, categorical effects, and no interactions were entered into the model due to the lack of data involved in interactions among fixed effects.

All applicable institutional and/or national guidelines for the collection, care and use of animals were followed. Field and laboratory procedures were carried out under the permission of the competent authorities *Consejería de Medio Ambiente de Madrid* (permits 10/064263.9/15, 10/072229.9/16, 10/132950.9/17, 10/007480.9/18, 10/084888.9/18) and *Instituto Aragonés de Gestión Ambiental* (500201/24/2015/226).

## Results

### Ex situ experiment

**Fig 2:**
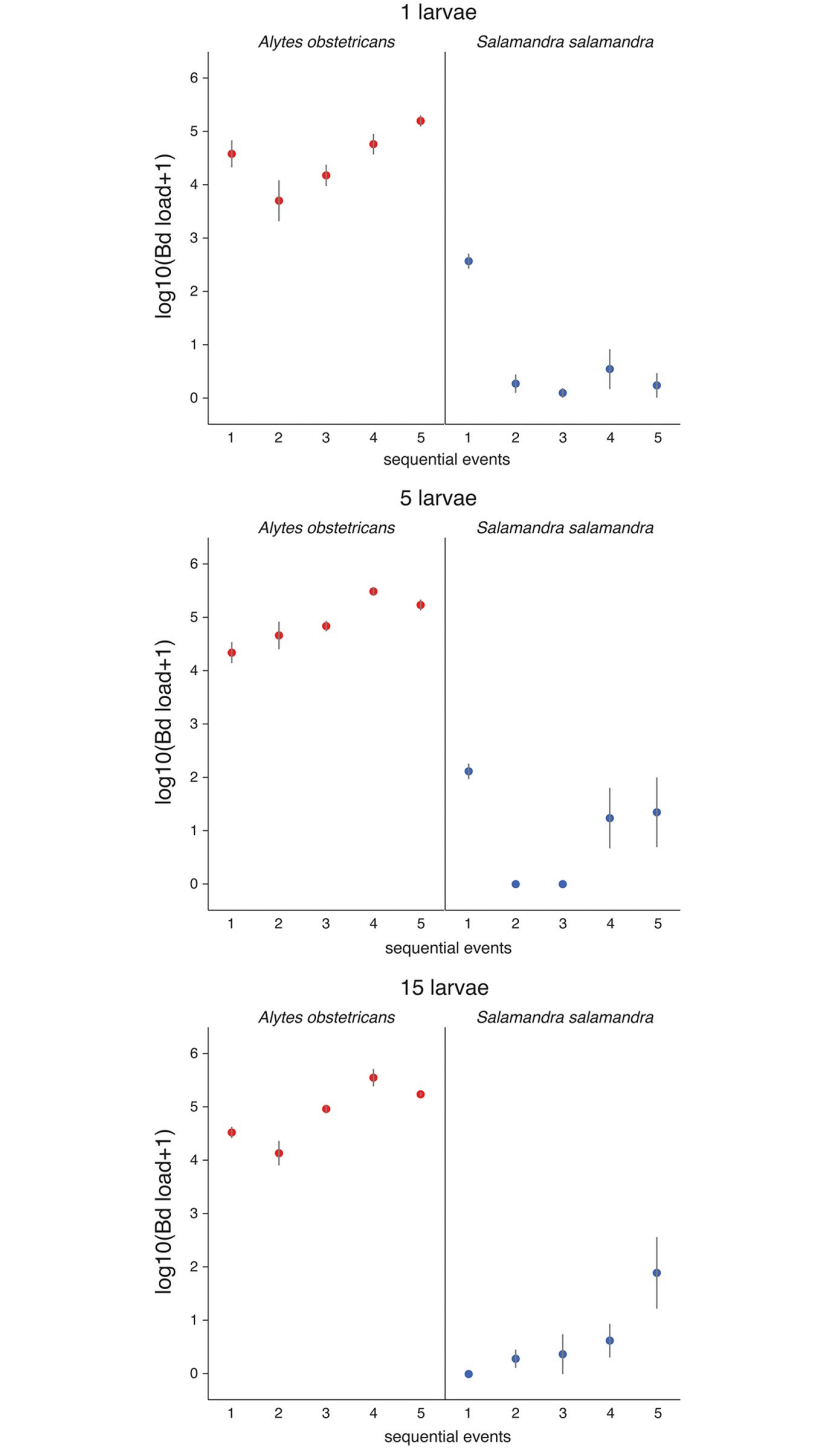
*Bd* loads of midwife toads (*Alytes obstetrica*ns) and fire salamanders (*Salamandra salamandra*) in the *ex situ* experiment on infection of the impact of host density (one, five and fifteen larvae) and time elapsed (five sequential events from 1 to 5 of repeated sampling during five months). Vertical bars depict mean ± one standard error of *Bd* load in decimal logarithm.

The within-subjects mixed model including the full set of interaction terms was the best model (AICc = 425.0; main effects AICc = 461.6; null AICc = 592.0) and was significant (likelihood ratio test of the model compared to the null model without fixed predictors: 184.2, *P*≪0.001). Fixed effects alone accounted for a high proportion of variance in *Bd* load (87.1%). Water temperature was positively and significantly related to *Bd* load (Table 1) and one Celsius degree increase in average water temperature led to a x1.29 increase in *Bd* load (regression coefficient of temperature: 0.112, se = 0.023; 10^0.112 = 1.294). Interspecific differences in *Bd* load were also significant (mean ± standard error in decimal logarithm: midwife toads 4.73 ± 0.089, fire salamanders 0.79 ± 0.091). Larvae density had, on average, a subtle positive influence on *Bd* infection (low figure of partial-eta^2^ in Table 1), as the experimental group of larvae containing only one animal did generate weaker infection loads than those in the group with the greatest density. The sequential timing of “sampling events”, on average, did have any influence on *Bd* load. Nevertheless, the global effects of larvae density and “sampling events” were not generalizable, as all the interaction terms containing these effects were significant, with a prominent effect of the differences between species throughout the sequential sampling events. Thus, the main effects of density and time after the beginning of trials should be interpreted separately for the two species (Fig 2).

**Table 1:** Results of the within-subjects mixed model examining the influence of species identity (midwife toads vs fire salamanders), larval density (one, five and 15 animals per replicate), sampling events (five sampling dates) and water temperature on *Bd* infection intensity (in decimal logarithm). The same individual per tank was sampled in five different occasions with a time lag of ca. 30 days. SS: sum of squares. partial-η: partial-eta2 estimating relative magnitude effects.

	SS	partial-η	df	F	P
Temperature	14.19	0.129	1, 135	23.94	≪0.001
Species (midwife toad)	11.24	0.105	1, 165	18.96	≪0.001
Density	6.61	0.062	1, 165	11.15	0.001
Sampling events	0.09	0.002	1, 135	0.15	0.700
Species x Density	9.43	0.090	1, 165	15.92	≪0.001
Species x Sampling events	18.84	0.165	1, 135	31.79	≪0.001
Density x Sampling events	10.35	0.095	1, 135	17.46	≪0.001
Species x Density x Sampling events	8.02	0.078	1, 135	13.54	≪0.001

Midwife toads maintained high *Bd* loads throughout the experiment, with some evidence of increasing loads in some treatments (Fig 2). Fire salamander infections were weaker and decreased from the first measurement in the one and five larvae density treatments, while *Bd* load increased from the first to the fifth “sampling event” in the high density treatment (Fig 2). Moreover, *Bd* load of individually housed fire salamanders decreased until infections were almost undetectable in this treatment (Fig 2).

### Field trial experiment

**Fig 3:**
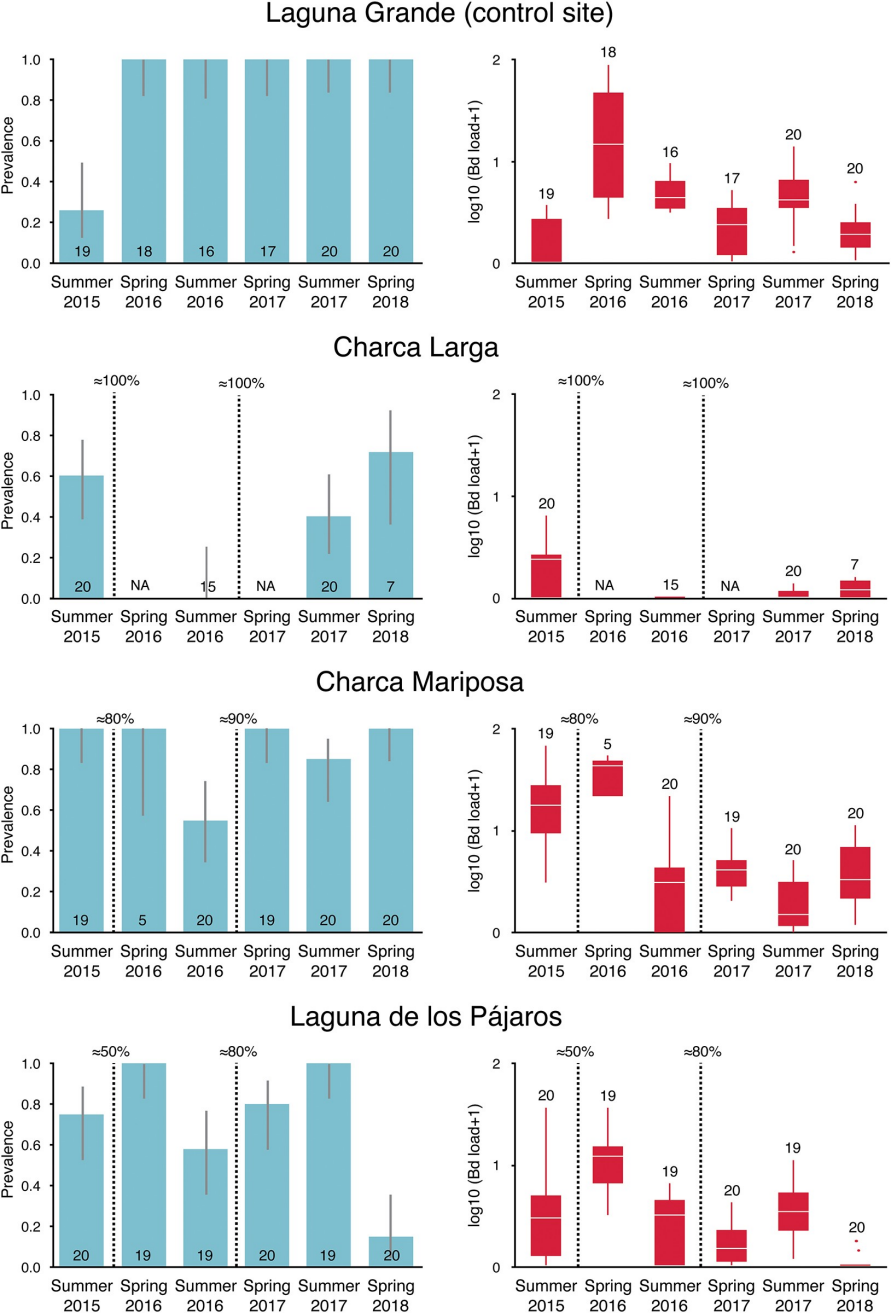
Pond specific changes in prevalence (left, vertical lines are 95% confident intervals) and load (right, in log transformed ZE values; horizontal lines depict medians, boxes represent interquartile ranges, whiskers extend to minima-maxima and dots show potential outliers) of *Bd* in salamander larvae (numbers above each bar or box-plot are sample sizes; *n* = 392 for both prevalence and infection intensity) within the Guadarrama National Park, Central Spain, 2015–2018. Dashed vertical lines indicate date (the end of the summer of the previous year and/or the early spring of the current year) and the approximate total proportion of larvae removed. Laguna Grande acted as control pond where no larvae were removed.

*Bd* loads of 392 salamander larvae were used to build the model (see Fig 3 for prevalence and load values by site and season), where fixed factors explained 26.2% of variance and both fixed and random effects explained 45.9%, yielding significant results for year (21.8% of the variance accounted for year accounting for the total variance observed among larvae and ponds, *F*_3,391_ = 44.8, *P<*<0.0001), season (5.9%, *F*_1,395_ = 36.4, *P<*<0.0001) and removal activity (4.0%, *F*_1,340_ = 24.3, *P<*<0.0001). As expected [43], *Bd* load was greater in spring than in summer (Fig 3). Load was weaker in ponds where larvae were removed the previous year after controlling for the other sources of variation, but only at Charca Larga and Charca Mariposa where we removed more than 80% of larvae at each removal. Conversely, and again after controlling for the other sources of variation, load of infection was even higher at Laguna de los Pájaros after larvae were removed the previous year.

## Discussion

A consistent role for temperature in *B*. *dendrobatidis* infection dynamics has been reported for this study system and for both of our study species [38,43,54–56]. The result of our *ex situ* experiment also suggests a significant effect of environmental temperature, albeit a weak one, on increasing load. The relative weak impact of temperature on load can be attributed to the low temperatures animals experienced over the 5 months of the experiment (< 10°C): Bosch et al. [54] showed that shifts in disease in *A*. *obstetricans* attributable to changes in infection dynamics were associated with temperatures approximating optimal, *ex situ* growth conditions for *Bd* (approx. 17–24°C).

Reducing host density is frequently mentioned as a potential mitigation strategy for controlling infectious diseases of wildlife. Long-term surveys indicated that when infection loads are lower, survival and recruitment of susceptible species like *S*. *salamandra* are enough to allow population persistence [38]. Our *ex situ* experiment demonstrated a significant role of reduced density in *Bd* infection intensity at very low densities, and our field experiment showed that reducing the abundance of salamander larvae in the field had a significant impact on load of infection the following season. Recent studies indicate [57,58], self-reinfection is more important than contact with other infected hosts or the environment, however the *ex situ* component of our study does not support this, as the animals in the treatment entirely reliant on re-infection (individually housed animals) often were unable to maintain infections over the course of the experiment. We are aware, however, that the focal species in our field study (*S*. *salamandra*) may not play as significant role in maintaining infection than the previous reservoir in the system (*A*. *obstetricans*) played [37–39], as supported by our *ex situ* comparisons across species. Consequently, future research examining the effect of removals of species that function more clearly as interspecific reservoirs and super-spreaders could yield other outcomes.

Most studies of culling programs for wildlife have found that removal rates often need to be extremely high [59–62]. To control chronic wasting disease in reindeer, eradication was required [63,64]. Our field trial experiment achieved removal rates of 80–100% in just one or two sessions with less than 10 people involved and in relatively large ponds (>5000 m^2^). However, our results indicate that even significant reductions of host abundance produce weak impacts on load of infections. This result aligns with some recent work that illustrate how culls rarely achieved the goal of reduced prevalence, load or disease, especially for well-established enzootic diseases or when disease can persist in the environment [65–67]. In our study site, chytridiomycosis has been present for at least 20 years, whereas *Bd* persistence in the environment has never been recorded in these oligotrophic glacial ponds (Bosch, unpublished results). Across annual cycles, adult amphibians did not consistently sustain infections but instead gained and lost infections from year to year [68]. We also note that the two ponds where removals had the greatest impact were also the smallest in our system, and where we counted fewer than two individuals per shoreline meter during removals. In contrast counts of larvae at the larger ponds were more than twice that (4.4 individual/m). Therefore removal effort, pond size and initial density are confounded in our study and we cannot unambiguously ascribe cause to the effect of infection reductions. What is less ambiguous are year effects, which were strongest and affected all ponds, including our control pond, Laguna Grande, where larvae were not removed.

Although our *ex situ* experiment argues for a positive relationship between load and water temperature below 10°C, long-term monitoring data supports a negative relationship for fire salamander infections when air temperatures exceed 20°C [38]. The Peñalara Massif is experiencing atypical patterns of warming and it is plausible that the year-on-year effects that predominantly affected load in *S*. *salamandra* larvae during the course of this study were driven by continued increases in average summer temperatures [38]. These effects may be direct on the pathogen or may affect load estimates by accelerating larval development and removing older, overwintering larvae that are predominantly responsible for detectable infections in Peñalara data sets as they move from the aquatic to the terrestrial environment. Additionally, stochastic variation can play an important role in disease dynamics of wildlife [69] and may have played a role in our removal trials experiment. In conclusion, based on the weak evidence that larval removal is a successful management strategy, artificial reductions in host abundance may not be a recommendable option for mitigating chytridiomycosis in the field unless extremely large reductions are possible.

## Supporting information

S1 Data (XLSX)
